# A systematic review of transcranial magnetic stimulation treatment for autism spectrum disorder

**DOI:** 10.1016/j.heliyon.2024.e32251

**Published:** 2024-05-31

**Authors:** Li-Xia Yuan, Xing-Ke Wang, Chen Yang, Qiu-Rong Zhang, Sheng-Zhi Ma, Yu-Feng Zang, Wen-Qiang Dong

**Affiliations:** a School of Physics, Zhejiang University, Hangzhou, China; b Center for Cognition and Brain Disorders, The Affiliated Hospital of Hangzhou Normal University, Hangzhou, China; c Institute of Psychological Sciences, Hangzhou Normal University, Hangzhou, Zhejiang, China; d Zhejiang Key Laboratory for Research in Assessment of Cognitive Impairments, Hangzhou, Zhejiang, China; e TMS Center, Deqing Hospital of Hangzhou Normal University, Deqing, Zhejiang, China

**Keywords:** Autism spectrum disorder, Transcranial magnetic stimulation, Stimulation targets, Intervention efficiency

## Abstract

Autism spectrum disorder (ASD) is a behaviorally defined complex neurodevelopmental syndrome characterized by persistent social communication and interaction deficit. Transcranial magnetic stimulation (TMS) is a promising and emerging tool for the intervention of ASD by reducing both core and associate symptoms. Several reviews have been published regarding TMS-based ASD treatment, however, a systematic review on study characteristics, specific stimulating parameters, localization techniques, stimulated targets, behavioral outcomes, and neuroimage biomarker changes is lagged behind since 2018. Here, we performed a systematic search on literatures published after 2018 in PubMed, Web of Science, and Science Direct. After screening, the final systematic review included 17 articles, composing seven randomized controlled trial studies and ten open-label studies. Two studies are double-blind, while the other studies have a moderate to high risk of bias attributing to inadequate subject- and evaluator-blinding to treatment allocation. Five studies utilize theta-burst stimulation mode, and the others apply repetitive TMS with low frequency (five studies), high frequency (six studies), and combined low and high frequency stimulation (one study). Most researchers prioritize the bilateral dorsolateral prefrontal lobe as stimulation target, while parietal lobule, inferior parietal lobule, and posterior superior temporal sulci have also emerged as new targets of attention. One third of the studies use neuronavigation based on anatomical magnetic resonance imaging to locate the stimulation target. After TMS intervention, discernible enhancements across a spectrum of scales are evident in stereotyped behavior, repetitive behavior, and verbal social domains. A comprehensive review of literature spanning the last five years demonstrates the potential of TMS treatment for ASD in ameliorating the clinical core symptoms.

## 1 Introduction

Autism spectrum disorder is a complex neurodevelopmental disorder characterized by persistent social communication and interaction deficit, as well as stereotyped behaviors, interests, and activities [1]. Since Victor Lotter's pioneering epidemiological study, the prevalence of ASD has steadily increased. Currently, ASD affects approximately 1 % of the global population, predominantly impacting men and presenting comorbidities in over 70 % of cases [2]. Treatments for ASD patients generally involve behavioral and drug approaches. Behavioral training is the most effective intervention; however, it is a highly expensive and time-consuming approach. On the other hand, currently no biomedical treatment for core symptoms of ASD has been established [3]. This means the efficacy of drug treatment is limited to managing concomitant symptoms and is associated with side effects, unable to address the underlying core symptoms of ASD [2,4].

Given the aberrant synaptic plasticity and excitation/inhibition ratio in ASD and the capacity of transcranial magnetic stimulation (TMS) to modulate cortical excitability and plasticity, the potential for TMS in the field of ASD research is beginning to be explored in laboratories world-wide [1]. As TMS is believed to induce lasting change in the brain by altering the mechanism of neural plasticity [5], some have postulated that TMS may be able to normalize social and cognitive performance in ASD by stabilizing aberrant neuroplasticity [6]. Moreover, two recent meta-analyses found that TMS can improve the core symptoms of ASD [7,8]. Barahona-Correa et al., conducted a systematic review and meta-analysis of 23 studies using TMS to treat ASD, and found that TMS had significant but moderate effects on repetitive behavior, social behavior, and executive function [7]. A meta-analysis of 12 studies by Smith et al. also showed that TMS improved social withdrawal, stereotyped behavior, and executive function in adolescents with ASD and an intelligent quotation greater than 65 [8]. The collective findings highlight the promising potential of TMS in the treatment of ASD. Additionally, TMS is non-invasive and considered relatively safe and well tolerated for ASD, even in the pediatric population [1]. According to a recent review, the overall prevalence of reported adverse effect of TMS among ASD was 25 %, including headache, facial discomfort, irritability, pain at the application site, and headedness or dizziness [9].

Several reviews have been published regarding TMS-based ASD treatment [1,8,10,11]. Casanova et al., reviewed the changes in behavior outcomes of studies using TMS to treat ASD before 2015 [10]. Oberman et al. focused on the application of TMS for ASD as both an investigational and therapeutic tool [1]. In 2020, Huashuang et al. conducted a systematic review and meta-analysis of 11 studies prior to 2020 to understand the safety and tolerability of TMS in ASD [9]. In 2020, Casanova et al., reviewed the neuropathologic underpinning of TMS for ASD treatment regarding the cortical inhibitory imbalance characterized by gamma oscillations in electroencephalogram (EEG) signal and its correlation with clinical outcomes [11]. In 2022, Smith et al. performed a new review by limiting the frequency, intelligence quotient, and stimulating brain region, examining 12 studies using low-frequency TMS or TBS to treat high-functioning ASD by targeting the left dorsolateral prefrontal cortex (DLPFC) published from 2009 to 2021 [8]. In 2018, Barahona-Correa et al., completed a systematic review on TMS for ASD treatment regarding study design, TMS modality, recruited subjects, study quality, stimulation targets, stimulation parameters, outcome measures [7]. However, there has been no updated systematic review on these aspects since 2018.

As early as 2005, Theoret et al. have begun to use TMS as an exploratory tool in the ASD community, opening a new avenue for investigating the neural mechanisms underlying the condition [12]; In 2009, Sokhadze et al. took this a step further by experimenting with TMS as an intervention in ASD populations [13]. However, TMS-based ASD treatment began to evolve more noticeably since 2018. For example, the protocol of the trial, target brain region and positioning tools, and stimulation pattern. Specifically, in addition to the bilateral DLPFC, the stimulated brain regions are more diverse, with areas such as the temporoparietal junction (TPJ), posterior superior temporal sulci (pSTS), and inferior parietal lobule (IPL) emerging as new target regions [[14], [15], [16]]. Secondly, intermittent Theta Burst Stimulation (iTBS) and continuous Theta Burst Stimulation (cTBS) stimulation mode has become increasingly favored by researchers for ASD treatment. Thirdly, the study quality has increased. For example, the prevalence of randomized controlled trials and the number of participants has increased dramatically over the years [11,17]. Large samples encompassing different age ranges and levels of functioning is beneficial towards the validity and reliability of TMS for ASD treatment. Thus, a timely review is necessary and can provide a summary for researchers involving recent studies of transcranial magnetic stimulation for ASD treatment.

In the current study, we focused on literatures using TMS to treat ASD between 2018 and 2023 and systematically reviewed study characteristics, specific parameters of TMS, localization techniques and stimulus targets, behavioral outcomes, and neuroimage biomarker changes with reference to PRISMA2020 [18]. Furthermore, the problems and challenges and their potential solutions are discussed. This study will facilitate to understanding the progress and difficulties of the research on the use of TMS in the treatment of autism.

## 2 Methods

### 2.1 Eligibility criteria

Types of studies: we included studies that were written exclusively in English, and published between January 1st, 2018 and June 5th, 2023, including randomized controlled trials, controlled trials, parallel trials, single-blind trials, and double-blind trials.

Types of subjects: Patients of any age or sex with a primary clinical diagnosis of ASD based on the Diagnostic and Statistical Manual of Mental Disorders, Fifth Edition (DSM 5), DSM 4, Autism Spectrum Quotient (AQ), ASD Diagnostic Observation Schedule-2 (ADOS-2) or the clinician's judgment were included.

Types of interventions: the current study focuses exclusively on TMS intervention. It is worth noting that TMS may have been administered as a standalone treatment or in combination with other interventions, including standard care, which may or may not have a confounding effect on TMS intervention treatment quality.

Types of outcome measures: 1) characteristics of included studies, such as number of subjects, type, age, sex, IQ, and type of experiment. 2) TMS parameters including treatment course, frequency of intervention, pattern, frequency intensity, pulses each time, trains, intervals, and session duration. 3) settings related to stimulation target including target region, locating method, positioning tool, and position/coordinates. 4) behavioral measurement changes after TMS treatment. 5) neuroimaging biomarker changes for ASD after TMS treatment.

### 2.2 Information sources

We conducted electronic searches for eligible studies from January 1st, 2018 to June 5th, 2023 within PubMed, Web of Science, and Science Direct.

### 2.3 Search strategy

**Fig. 1:**
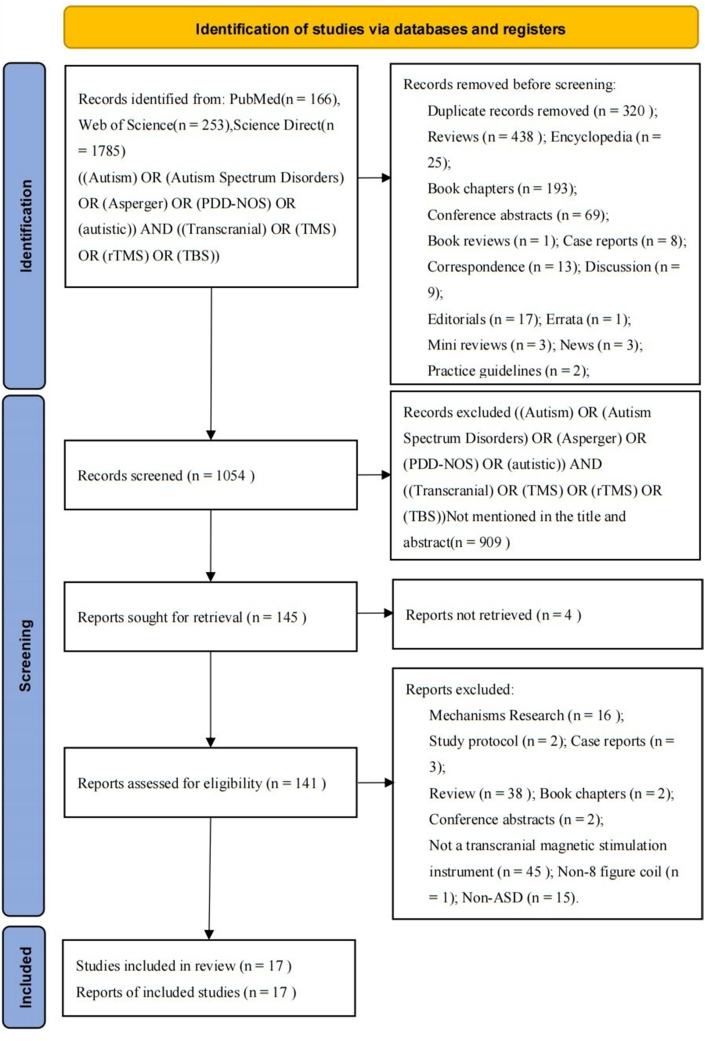
Preferred reporting items for systematic reviews and meta-analyses (PRISMA) flow diagram for study selection.

As shown in Fig. 1, the advanced search Boolean operators AND OR were used to run the search: ((Autism) OR (Autism Spectrum Disorders) OR (Asperger) OR (PDD-NOS) OR (autistic)) AND ((Transcranial) OR (TMS) OR (rTMS) OR (TBS)).

### 2.4 Selection process

**Fig. 2:**
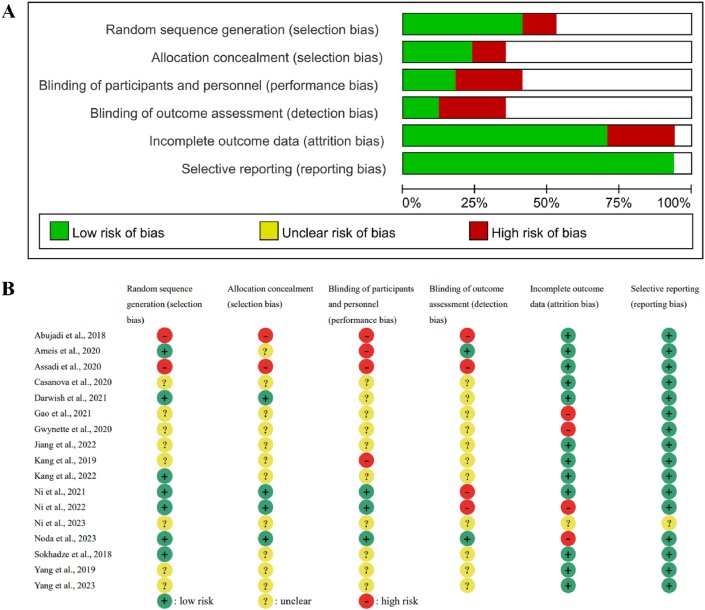
Graph and summary of bias graph. A: Risk of bias graph: review authors' judgements about each risk of bias item presented as percentages across all included studies. B: Risk of bias summary: review authors' judgements about each risk of bias item for each included study.

As outlined in Fig. 1, following PRISMA 2020 protocol, in the first step, 166, 253, and 1785 results were retrieved using keywords from three databases: PubMed, Web of Science, and Science Direct. In the second step, the retrieved information, such as title, author, abstract, publication date and impact factor, was imported into the table tool. According to the document type marked in the Science Direct database, 320 duplicate records were deleted. Next, types of articles that did not meet the requirements such as letters and books were deleted using the database marking function. These include 438 reviews, 25 encyclopedias, 193 chapters in books and 13 abstracts of meetings, as well as 1 book review, 8 cases, 13 correspondence, 9 discussions, delete 17 reviews, 1 erratum, 3 mini reviews, 3 news, 2 practice guides, 28 brief correspondence, and 20 others. The third step was to use keyword search in the title and abstract of the remaining results, deleting 909 results that did not contain the relevant keywords. The fourth step was to further check the article to ensure that the full text can be reviewed and downloaded. During the fifth step we read the full text further and deleted 16 mechanism studies, 2 trial protocols, 3 cases, 38 reviews, 2 chapters in books, 2 conference abstracts, 45 articles that did not use the 8-word transcranial magnetic stimulation instrument as a research tool, 1 article that did not use the figure- 8 coil, and 15 articles unrelated to ASD. Finally, we used the revman5 software (https://revman.cochrane.org/info) to evaluate the quality of the remaining 17 studies; the results of which are shown in Fig. 1, Fig. 2.

### 2.5 Data collection process

A researcher designed a data extraction form that was used to extract data from eligible studies, and the review was independently verified by three people.

### 2.6 Data items

The collected data includes following items: 1) study characteristics: number of subjects, type, age, sex, IQ; 2) experimental scheme: type of experiment, frequency of intervention, mode of stimulation, specific parameters, localization methods and tools, brain regions and locations of stimulation; 3) research results: changes in major behavioral outcomes, changes in assessment scales and scores used, 4) changes in functional connectivity and EEG outcomes.

## 3 Results

### 3.1 Characteristics of included studies using TMS for ASD treatment

Table 1 provides a comprehensive overview of 17 studies conducted between 2018 and 2023 that employed TMS as a therapeutic approach for ASD. The table includes information on the ASD subtype, research type, number of subjects, age, and IQ. Regarding the subtypes of ASD individuals, four studies focused on subjects without intellectual disabilities (ID), four studies on subjects with ID, one study included subjects with IQ greater than 50, and the other studies did not give information about subjects’ IQ. In contrast, seven studies did not furnish specific details pertaining to the classification of their subjects. Furthermore, a significant majority of the subjects in the examined studies were male, with only one study incorporating a small sample of five female participants [19]. The age range of the subjects varied from 2 years to 30 years [20,21].

**Table 1:** Characteristics of included studies using transcranial magnetic stimulation for autism spectrum disorder treatment.

literature	criteria	ASD subtype	No. of subjects/males	age (years)	IQ	research type
Ni et al., 2023	DSM 5/ADOS-2	with ID	60	8–30	<70	randomized, double-blind sham-controlled
Ameis et al., 2020	ADOS-2/DSM 4/DSM 5	without ID	40/28	22.6 ± 4.5	>70	randomized, double-blind, sham-controlled
Ni et al., 2022	DSM 4/DSM 5	–	13/11	22.7 ± 1.4	–	randomized, single-blind, sham-controlled crossover
Ni et al., 2021	DSM 4	without ID	73/--	8–17	≥70	randomized, single-blind, sham-controlled + open label
Darwish et al., 2021	–	–	30/20	3–10	–	randomized, sham-controlled
Kang et al., 2022	DSM 5	with ID	16/13	7.8 ± 2.1	<70	randomized, sham-controlled
Sokhadze et al., 2018	DSM 4/DSM 5	without ID	106/87	13.1 ± 1.8	>80	randomized, waitlist controlled
Kang et al., 2019	DSM 5	with ID	32/26	7.8 ± 2.1	–	waitlist controlled
Gwynette et al., 2020	DSM 5	with ID	10/9	23–29	<60	open-label
Yang et al., 2019	DSM 5/ADIR/ABC	with ID	11/7	3–12	<70	open-label
Casanova et al., 2020	DSM 4/DSM 5	without ID	19/14	14.4 ± 3.6	>80	open label
Abujadi et al., 2018	–	–	10/10	9–17	>50	open-label
Yang et al., 2023	DSM 5	–	24/3	8.04 ± 3.54	–	open-label
Jiang et al., 2022	DSM 5	–	24/3	8.04 ± 3.54	–	open-label
Assadi et al., 2020	ADOS-2	–	4/4	11–17	–	open-label
Noda et al., 2023	DSM 5	–	18/13	41.8 ± 11.9	–	open-label
Gao et al., 2021	DSM 5	–	39/31	2–18	–	open-label

ID: Intellectual disability; ADOS-2: Autism Diagnostic Observation Schedule; DSM: The diagnostic and statistical manual of mental disorders; ABC: Autism Behavior Checklist; ADIR: Autism Diagnostic Interview-Revised; IQ: Intelligence Quotient.

Among the 17 included articles, seven were randomized controlled trials (RCTs), and the remaining ten were categorized as open-label studies. It is essential to acknowledge that a considerable number of the reviewed studies exhibited a moderate to high risk of bias (Fig. 2(A and B)), primarily attributed to the absence of subject and evaluator blinding in the treatment assignment. Notably, only two of the studies were identified as double-blind, indicating a potential influence on the study outcomes due to the lack of blinding. Additionally, it is noteworthy that three studies originated from the same research group at Chang Gung Memorial Hospital in Linkou, three studies were conducted by researchers at Capital Medical University, and two studies were affiliated with Hebei University. Furthermore, two of the studies utilized the same sample population [22,23].

### 3.2 Parameters for the treatment of ASD with TMS

Table 2 demonstrated the diversity in TMS application strategies for ASD within the existing literature. Among the 17 reviewed articles, seven studies reported conducting the TMS intervention five times per week [24], indicating its prevalence as the most frequently employed treatment frequency. Conversely, other studies opted for alternative frequencies, such as three times a week (in one article), twice a week (in four articles), or once a week (in two articles). The total duration of interventions varied widely, spanning from 1 to 18 weeks across different studies. Notably, one study failed to report both the number of treatments per week and the overall intervention duration [25].

**Table 2:** Parameters for the treatment of ASD with TMS.

literature	treatment course/frequency	pattern	frequency	intensity	pulses each time	trains	intervals	duration
Kang et al., 2019	9 weeks/twice per week	rTMS	1 Hz	90 % MT	180 pulses	18	20 s	540 s
Casanova et al., 2020	18 weeks/1 time per week	rTMS	1 Hz	90 % MT	180 pulses	9	20–30 s	–
Darwish et al., 2021	4 weeks/5 times per week	rTMS	1 Hz	70 % MT	1800 pulses	20	20 s	40 min
Sokhadze et al., 2018	6, 12, 18 weeks/1 time per week	rTMS	1 Hz	90 % MT	180 pulses	9	20–30 s	–
Kang et al., 2022	9 weeks/twice per week	rTMS	1 Hz	90 % MT	180 pulses	18	20 s	540 s
Gwynette et al., 2020	25 days/1 time per day	rTMS	10 Hz	120 % RMT	3000	60	10 s	900 s
Assadi et al., 2020	3 weeks/3 times per week	rTMS	10 Hz	80 % MT	1000 pulses	10	20 s	5 min
Yang et al., 2023	3 weeks/5 times per week	rTMS	15 Hz	50 % stimulator output	375 pulses	5	10 min	2425 s
Jiang et al., 2022	3 weeks/5 times per week	rTMS	15 Hz	–	375 pulses	5	10 min	2425 s
Yang et al., 2019	3 weeks/5 times per week	rTMS	20 Hz	50 % stimulator output	500 pulses	5	10 min	3025 s
Ameis et al., 2020	4 weeks/5 times per week	rTMS	20 Hz	90 % MT	1500 pulses	25	30 s	787.5 s
Gao et al., 2021	2 weeks/5 times per week	rTMS	rDLPFC: 1 Hz	25 % MT	rDLPFC: 896	rDLPFC: 28	rDLPFC: 1 s	rDLPFC: 924 s
			lDLPFC: 10 Hz		lDLPFC: 1440	lDLPFC: 45	lDLPFC: 10 s	lDLPFC: 594 s
Ni et al., 2023	8 weeks/twice per week	cTBS	50 Hz 3-pulse bursts at 5 Hz	90%AMT	600 pulses	200	200 ms	40 s
Noda et al., 2023	–	iTBS	50 Hz 3-pulse bursts at 5 Hz	120 % RMT	600 pulses	20	8 s	200 s
Abujadi et al., 2018	3 weeks/5 times per week	iTBS	50 Hz 3-pulse bursts at 5 Hz	100 % MT	900 pulses	30	8 s	300 s
Ni et al., 2022	5 day/1 time per day	iTBS	50 Hz 3-pulse bursts at 5 Hz	80 % AMT	600 pulses	20	8 s	300 s
Ni et al., 2021	4 weeks/twice per week	iTBS	50 Hz 3-pulse bursts at 5 Hz	80 % AMT	600 pulses	20	8 s	300 s

rTMS: repetitive transcranial magnetic Stimulation; iTBS: intermittent theta burst stimulation; RMT: resting motor threshold; MT: motor threshold; AMT: activity motor threshold; lDLPFC: left dorsolateral prefrontal cortex; rDLPFC: right DLPFC; cTBS: continuous TBS.

Two common modes of TMS are frequently applied, namely rTMS and iTBS. In general, iTBS is purported to facilitate cortical excitability and promote long-term potentiation like effects [26]. Among the 17 studies reviewed, the rTMS model was more prevalent, utilized in 12 of the studies. By convention, repetitive TMS of less than 1 Hz is considered low frequency stimulation. Models on long-term potentiation suggest that low frequency TMS is inhibitory while faster stimulation (≥5 Hz) is excitatory [27]. Specifically, five out of the 17 studies employed a low-frequency rTMS mode at 1Hz. In contrast, the high-frequency modes most commonly utilized were 10Hz, 15Hz, and 20Hz. The majority of the studies employed a single frequency for their intervention, except for one study that simultaneously utilized 1Hz and 10Hz frequencies to stimulate the DLPFC [20]. The objective of this particular study was to investigate the impact of TMS on the core symptoms of ASD and comorbid sleep problems, as well as to explore the potential mediating role of ASD symptoms in the relationship between rTMS intervention and sleep improvement.

### 3.3 Stimulation targets and localizing methods

Table 3 provides a comprehensive overview of localization tools, localization methods, stimulation brain regions, and detailed targets when using TMS to treat ASD. The currently employed localization tool encompass EEG camp, swimming cap, and neuronavigation based on T1 anatomical MRI. Remarkably, more than half of the studies utilized EEG caps for positioning, representing the predominant techniques utilized to date. Although Kang et al. and Gao et al. mentioned the use of EEG caps, they did not provide a detailed description of the positioning method employed [20,28].

**Table 3:** Tool and method for localizing stimulation target, stimulating brain regions, and detailed targets for the treatment of ASD with TMS.

literature	tool	method	stimulating brain regions	detailed targets
Gwynette et al., 2020	EEG cap	Beam-F3 method	left DLPFC	F3
Noda et al., 2023	EEG cap	Beam-F3 method	left DLPFC	F3
Yang, 2023	EEG cap	Electrode P3	left PL	P3
Jiang, 2022	EEG cap	Electrode P3	left PL	P3
Yang et al., 2019	EEG cap	Electrode P3	left IPL	P3
Darwish et al., 2021	EEG cap	Electrode F5	left IPL	F5
Kang et al., 2022	EEG cap	–	bilateral DLPFC	–
Gao et al., 2021	EEG cap	–	bilateral DLPFC	–
Kang et al., 2019	EEG cap	5 cm	bilateral DLPFC	–
Casanova et al., 2020	swimming cap	5 cm	bilateral DLPFC	–
Sokhadze et al., 2018	swimming cap	5 cm	bilateral DLPFC	F3 and F4
Ameis et al., 2020	neuronavigation	T1 anatomical MRI	bilateral DLPFC	±50, 30, 36
Ni, 2023	neuronavigation	T1 anatomical MRI	left DLPFC	42.5, 31.7, 41.4
Assadi et al., 2020	neuronavigation	T1 anatomical MRI	left IPL	–
Abujadi et al., 2018	neuronavigation	T1 anatomical MRI	right DLPFC	–
Ni et al., 2022	neuronavigation	T1 anatomical MRI	bilateral pSTS	±50.5, 57.1, 7.9
Ni et al., 2021	neuronavigation	T1 anatomical MRI	bilateral pSTS	–

DLPFC: dorsolateral prefrontal cortex; IPL: inferior parietal lobule; TPJ: temporoparietal junction; pSTS: posterior superior temporal sulcus; Explanation: the coordinates in the rightmost column are Montreal Neurological Institute spatial coordinates; left PL: left parietal cortex.

Within the realm of targeted brain regions, the DLPFC has emerged as the most favored area of interest in the past five years. Among the ten studies that stimulated the DLPFC, six targeted the bilateral DLPFC. However, Gwynette et al. exclusively stimulated the left DLPFC of the subjects, while Abujadi et al. applied 15 sessions of iTBS specifically over the right DLPFC [29,30].

Three studies employed an identical stimulation sequence [11,19,28,31]. Notably, the first six interventions targeted the left DLPFC, the subsequent six focused on the right DLPFC, and the final six reverted to the left DLPFC. Conversely, the remaining 7 articles did not provide reference positions or specific coordinates for the electrode cap; they merely indicated the name of the stimulated brain area.

These diverse approaches in localization methods and stimulation targets underscore the ongoing exploration and utilization of various methodologies in TMS studies for ASD treatment.

### 3.4 Intervention effect on behavior of TMS for ASD

Table 4 provides a comprehensive summary of the scales utilized in the study, along with the corresponding baseline, post-intervention, and follow-up scores. A diverse set of 21 different scales was employed across the studies, and it is noteworthy that each study utilized multiple scales concurrently.

**Table 4:** Used scales and corresponding score changes after TMS for ASD treatment.

literature	scales	score changes
Gwynette et al., 2020	HAM-D17	22 to10
	SRS-2	73.5 to 73.5
	RAADS-R	36 to 38
	RBS-R	55 to 20
	ABC	56 to 20
Yang et al., 2019	ATEC: language	16.1 to 9.8
	ATEC: social	19.8 to 12.2
	ATEC: sensory and cognitive awareness	21.3 to 19.2
	ATEC: health and behavioral problems	21.2 to 18.6
	VerBAS	30.6 to 38.6
Kang et al., 2019	ABC total	62.06 to 52.19
	ABC: Sensory behavior	13.31 to 11.94
	ABC: Social relating	15.12 to 11.06
	ABC: Body and object use	9.94 to 8.31
	ABC: Language and communication	11.75 to 9.31
	ABC: Social and adaptive skills	11.88 to 11.13
Casanova et al., 2020	RBS-R: lethargy/social withdrawal scores	7.89 to 5.89
	RBS-R: stereotypy scores	5.53 to 3.26
	RBS-R: compulsive behavior scores	3.95 to 2.00
	RBS-R: total repetitive behaviors T-score	23.74 to 5.21
	ABC: irritability scores	11.74 to 6.63
	ABC: lethargy/social withdrawal scores	7.89 to 5.89
	ABC: hyperactivity scores	17.47 to 8.79
Ameis et al., 2020	BRIEF MCI	71.7 to 61.8
	SWM errors	23 to 19.3
Assadi et al., 2020	ADOS-2 total	15.75 to 12.50
	SRS-2 total raw	81.75 to 64.75
	D-KEFS	24.25 to 25.00
	D-KEFS category	23.25 to 29.75
	D-KEFS switching	11.75 to 10.75
	EVT-2 raw	116.00 to 118.50
Sokhadze et al., 2018	RBS-R	26.5 to 14.6
	RBS-R: ritualistic behavior rating	9.61 to 5.55
	RBS-R: stereotype behavior rating	5.71 to 2.73
	RBS-R: aberrant behavior checklist	12.39 to 6.38
	RBS-R: lethargy/social withdrawal scores	11.50 to 6.42
	RBS-R: hyperactivity scores	18.09 to 10.75
Yang, 2023	SRS	107.88 to 100.67 to 102.29 (4wl)
	RBS-R	25.63 to 19.71 to 20.96 (4wl)
	ATEC total score	69.67 to 60.17 to 64.46 (4wl)
	ATEC-language scale	12.13 to 10.29 to 11.58 (4wl)
	ATEC-social scale	20.88 to 16.92 to 17.79 (4wl)
	ATEC-sensory and cognitive awareness scale	17.00 to 15.54 to 16.42 (4wl)
	ATEC-health and behavioral problems scale	19.67 to 17.42 to 18.67 (4wl)
Jiang, 2022	SRS	107.88 to 100.67 to 102.29 (4wl)
	RBS-R	25.63 to 19.71 to 20.96 (4wl)
	ATEC total score	69.67 to 60.17 to 64.46 (4wl)
	ATEC-language scale	12.13 to 10.29 to 11.58 (4wl)
	ATEC-social scale	20.88 to 16.92 to 17.79 (4wl)
	ATEC-sensory and cognitive awareness scale	17 to 15.54 to 16.42 (4wl)
	ATEC-health and behavioral problems scale	19.67 to 17.42 to 18.67 (4wl)
Ni, 2023	SRS	109.3 to 95.5 to 94.0 (12wl)
	RBS-R	37.8 to 27.3 to 28.6 (12wl)
	EDI	30.6 to 24.6 to 25.8 (12wl)
	ABAS-II	77.1 to 80.2 to 81.3 (12wl)
	RMET, total correct	21.3 to 22.1 to 22.1 (12wl)
	RPM	87.3 to 88.0 to 89.8 (12wl)
	BRIEF	71 to 67.2 to 66.4 (12wl)
Abujadi et al., 2018	RBS-R	27.40 to 13.30
	Stroop	97.30 to 17.33
	WSCT	0.30 to 0.23
	YBOCS	11.80 to 8.50
Ni et al., 2022	ADOS: reciprocal social interaction	6.3 to 6.0 (active + sham)/6.9 (sham + active)
	ADOS: language and communication	3.8 to 3.8 (active + sham)/4.0 (sham + active)
	ADOS: stereotyped behaviors and restricted interest	1.2 to 1.2 (active + sham)/1.2 (sham + active)
	Total AQ-parents	27.6 to 23.8 (active + sham)/32.9 (sham + active)
	Total AQ-self	32.4 to 31.8 (active + sham)/30.9 (sham + active)
Kang et al., 2022	ABC total	reduced 10
	ABC: sensory behavior	reduced 2
	ABC: social relating	reduced 4
	ABC: body and object use	reduced 2
	ABC: language and communication	reduced 3
	ABC: social and adaptive skills	reduced 1
Ni et al., 2021	SRS	107.3 to 103.4, 98.5, and 97.2 for 4, 8, and 12-week stimulation
	RBS-R	32.2 to 28.9, 25.9, and 24.3 for 4, 8, and 12-week stimulation
	RMET	20.8 to 21.9, 21.8, and 22.0 for 4, 8, and 12-week stimulation
Noda et al., 2023	HAM-D 21	14.3 to 8.6
	MADRS	23.7 to 14.2

HAM-D17: Hamilton Rating Scale for Depression; SRS-2: Self-reported questionnaires Social Responsiveness Scale, Second Edition-Adult; RAADS-R: Ritvo Autism Asperger's Diagnostic Scale-Revised; RBS-R: Repetitive Behavior Scale-Revised; ABC: Aberrant Behavior Checklist; ATEC: Autism Treatment Evaluation Checklist; BRIEF MCI: Behavioral Rating Inventory for Executive Function Metacognition Index; SWM Errors: spatial working memory total errors score: ADOS-2: Autism Diagnostic Observation Schedule-2nd edition; D-KEFS: Delis-Kaplan Executive Function System; EVT-2: Expressive Vocabulary Test-2nd Edition; CARS: Childhood Autism Rating Scale; VerBAS: The verbal behavior assessment scale; WSCT: Wisconsin Card Sorting Test; YBOCS: Yale Brown Obsessive Compulsive Scale; SDQ: Strengths and Difficulties Questionnaire; RMET: Reading the Mind in the Eyes Test; MADRS: Montgomery-Asberg Depression Rating Scale; ABAS-II: Adaptive Behavior Assessment System-II; EDI: Emotion Dysregulation Inventory; RPM: Raven's Progressive Matrices.

#### 3.4.1 Repetitive behavior and stereotyped behavior

Regarding repetitive behavior, 8 studies measured improvements after TMS intervention. The Repetitive Behavior Scale-Revised (RBS-R) was the primary tool used to quantify changes in subjects’ behavior. The largest decrease in scores was 35 points [30], while the smallest change was 7.9 points [32]. Of the 8 studies, only Ni et al. stimulated the bilateral posterior superior temporal sulcus (pSTS). The target stimulation area in the other four studies was the DLPFC.

Three studies reported changes in stereotyped behavior after TMS treatment. Casanova et al. documented a decrease in stereotype scores from 5.53 to 3.26 [11]. Similarly, Sokhadze et al. reported an even greater decrease from 5.71 to 2.73 [17]. However, in Ni et al. where 13 adult autistic patients had their bilateral pSTS stimulated with iTBS five times, the scores of stereotyped behaviors and restricted interests did not change [16].

#### 3.4.2 Language and social behavior

Ten studies investigated the impact of TMS intervention on language and social scale scores, employing scales such as ATEC, SRS-2, and ABC. Among them, six studies reported significant improvements in language and social behavior, as evidenced by decreased scores on the respective scales. Notably, Ni et al.'s study demonstrated contrasting results, where they administered five iTBS sessions to the bilateral pSTS in 13 autistic patients. In this study, the interactive social interaction score increased from 6.3 to 6.9 when the sham operation was performed first and then followed by stimulation of the pSTS. Additionally, the language and communication score increased from 3.8 to 4.0 [16].

### 3.5 Intervention effect on neuroimaging biomarker of TMS for ASD

Table 5 provides a comprehensive summary of nine neuroimaging biomarkers, along with specific changes observed before and after intervention. In addition to behavioral evaluations, nearly half of the articles reported EEG or task results. For instance, Kang et al. conducted 18 1Hz rTMS sessions of bilateral DLPFC in 32 patients with without ID autism, revealing significant increases in peak α frequency in the frontal, left temporal, right temporal, and occipital regions. Additionally, a significant increase in α coherence was observed in the central and right temporal regions [31]. In another study, Kang et al. reported significant differences in RR and DET between the experimental and control group [28]. Similarly, Casanova et al. observed significant differences in induced gamma oscillations in ASD subjects compared to neurotypical subjects before TMS [11]. Furthermore, Sokhadze et al. discovered that the TMS group had a lower right hemisphere P3a latency compared to the waiting list group and shorter latency to non-target Kanizsa disruptors for both the 12-week and 18-week TMS treatment groups [17]. Moreover, Jiang et al. reported hyper-variability in the resting-state networks of ASD patients and found that three-week rTMS treatment alleviated the hyper-fluctuations occurring in the frontal-parietal and frontal-occipital connectivity, contributing to the amelioration of ASD symptoms [22]. Additionally, Yang et al. observed that children with ASD exhibited significantly hypo-connected networks and sub-optimal network properties at both global and local levels compared to typically developing peers. After rTMS intervention, long-range intra- and inter-hemispheric connections showed a significant increase, especially within the alpha band. Moreover, network properties at both local and global levels improved substantially in the delta, theta, and alpha bands [23]. These findings highlight the potential of TMS intervention in influencing neuroimaging indicators and offer valuable insights into its effects on brain connectivity and functional changes in individuals with ASD.

**Table 5:** Neuroimaging biomarker changes for ASD after TMS treatment.

Literature	biomarker	brain area/stimuli	comparison	results
Sokhadze et al., 2018	amplitude of P100	parietal site	Post TMS vs. waitlist	lower
	latency of frontal P3a	frontal site	Post TMS vs. waitlist	shorter
	latency of parietal P3b	parietal site	Post TMS vs. waitlist	longer
	latency of frontal N100	frontal site	Post TMS vs. waitlist	longer
Kang et al., 2019	peak alpha frequency	frontal and occipital region, left and right temporal region	post vs. pre-TMS	significant increases
	alpha coherence	central lobe and right temporal lobe	post vs. pre-TMS	significant increase
Casanova et al., 2020	evoked gamma oscillations	task-irrelevant stimuli	post vs. pre-TMS	significant reduction
Kang et al., 2022	recursive rate	frontal, parietal, and occipital lobe, left and right temporal lobe	post vs. pre-TMS	significant differences
	deterministic	occipital lobe	post vs. pre-TMS	discernible difference
Yang et al., 2023	long-range connections	intra- and inter-hemispheric	post vs. pre-TMS	significantly promoted
	network properties	local and global	post vs. pre-TMS	greatly promoted
Jiang et al., 2022	hyper fluctuations of connectivity	frontal-parietal and frontal-occipital	post vs. pre-TMS	alleviated

rTMS: repetitive transcranial magnetic stimulation; pSTS: posterior superior temporal sulci.

## 4 Discussion

By systematically reviewing literatures using TMS to treat ASD since 2018, the study characteristics, specific parameters of TMS, localization techniques and stimulus targets, behavioral outcomes, and neuroimage biomarker changes were comprehensively summarized. Our result demonstrates that: 1) most studies have a moderate to high risk of bias; 2) TBS and rTMS are two commonly used stimulation modes; 3) bilateral DLPFC stands as the foremost targeted region, followed by PL, IPL, and pSTS, and EEG cap emerges as the primary tool for localization, followed by neuronavigation and swimming cap; 4) evident ameliorations in the clinical core symptoms manifest subsequent to TMS intervention; 5) only electroencephalogram is used to characterize the post-treatment neuroimaging changes.

### 4.1 Subject characteristics

Whether ASD should be seen as a kind of disease is still controversial, especially for without ID autism [33,34]. Some people believe that ASD is a kind of characteristic and suggested that ASD should be revised to Autism Related Disorder (ARD) in the diagnostic and statistical manual of mental disorders (DSM) [35]. However, it is also true that ASD has become a major global public health problem due to its complex condition, life-long symptoms, and lack of effective treatment, and brings heavy mental pressure and economic burden to families and society [36]. Whether ASD is classified as a disease or a characteristic, providing ways for improving the well-being (e.g., TMS) for ASD is very important. Moreover, more than 70 % of individuals with autism have concurrent medical, developmental, or psychiatric conditions [2,37], and treating the comorbidities of people with ASD is also critical.

The primary focus of researchers in the domain of ASD has predominantly been on without ID ASD cases, possibly due to the convenience of their higher intelligence quotient, which aligns better with experimental requirements, such as magnetic resonance imaging and TMS treatment [38]. However, it is essential to acknowledge that the criteria used to define without ID and without ID can vary significantly. In the included article, without ID subjects are characterized by an IQ < 70 [21], or IQ < 60 [30], while without ID subjects have an IQ > 80 [17], IQ > 70 [39], or IQ > 50 [29]. The inconsistent definitions pose a challenge for clarity and comparability.

Furthermore, the studies included in this review exhibited considerable variation in the number of subjects and the research designs employed. Some studies were conducted as control studies, while others followed the structure of randomized controlled trials (RCTs), including both double-blind and single-blind trials. It is important to recognize that non-RCT research designs are generally considered less reliable and are not directly comparable to RCTs. To enhance the robustness of the existing data, future studies on repetitive TMS therapy for ASD should prioritize using randomized, placebo-controlled, double-blind approaches, and should incorporate an adequate follow-up period after treatment to assess the long-term effects accurately [7].

Among the 17 articles reviewed, only one provided detailed information about ASD subtypes. The identification of ASD subtypes holds the potential to inform discussions regarding whether distinct treatment plans or specific TMS approaches should be tailored to address the unique characteristics of each subtype or core symptom. However, it is far from clear that whether different subtypes of ASD correspond to different treatments. Therefore, it is essential that future research on repetitive TMS therapy for ASD focuses on clarifying ASD subtypes or further exploring the heterogeneity within ASD [1,23]. Such efforts can lead to more personalized and effective therapeutic interventions for individuals with ASD.

TMS intervention for children and minors with ASD has both advantages and disadvantages. Early intervention may mean better prognosis, including alleviating the symptoms, reducing the family burden, benefitting general education, and facilitating a normal life [40]. However, children with ASD may be “labeled” when receiving treatment, leading to discrimination [35]. Therefore, protecting the mental health of children and minors with ASD is also very important during the therapeutic interventions.

### 4.2 TMS mode and parameters

Currently, the stimulation mode in the examined studies remains relatively fixed, yet the stimulation parameters exhibit significant variability, posing challenges for direct comparisons. In the iTBS mode, methods for measuring motor threshold, such as Active Motor Threshold (AMT) and Resting Motor Threshold (RMT), lack consistency across the studies. It is noteworthy that measuring motor thresholds in autistic patients presents difficulties due to their apprehension towards unfamiliar environments, equipment, and personnel, which could potentially interfere with the experimental process. To simplify the process, Yang et al. found that the RMT of children in the laboratory was mostly 40 %–50 %, so the stimulation intensity was uniformly set to 50 % of the output of the stimulator [14].

Another critical issue is determining the optimal treatment course for TMS in ASD management. Studies with a once-a-week frequency have demonstrated significant improvements in behavior and symptoms after an 18-week TMS treatment course [11,17]. Conversely, a treatment frequency of five times a week resulted in notable enhancements in core symptoms and sleep problems after only two weeks of TMS intervention. Additionally, Yang et al. (2019) reported significant reductions in language and social-related symptoms from pre-treatment to a 6-week follow-up after the second treatment course. The overall length of the treatment course also plays a crucial role, as suggested by Sokhadze et al. who found that treatment course length [17], such as 12 or 18 weeks, significantly influenced observed behavioral and ERP improvements in their trial involving autistic children, rather than the treatment frequency.

The relationship between treatment frequency, duration, and therapeutic effects remains unclear and appears to be highly individualized [7]. In future clinical practice, tailoring treatment plans according to the specific needs of each patient becomes imperative. This involves adjusting treatment duration and frequency to provide personalized and optimized intervention strategies for individuals with ASD undergoing TMS therapy. Such personalized approaches have the potential to enhance treatment outcomes and contribute to the overall effectiveness of TMS as an intervention for ASD.

Excitatory and inhibitory (E/I) imbalance (i.e., the increased E/I ratio) regarding the pathophysiological mechanisms in ASD is still disputed. Some studies illustrate that the individuals with ASD may have the E/I imbalance toward excitation as a result of reduced inhibition and/or increased excitation [41]. At the genetic level, the ASD have shown overexpressed excitatory glutamate receptors (NMDA and metabotropic glutamate receptor 5) and under-expressed inhibitory gamma aminobutyric acid A (GABAA) and B (GABAB) receptors [42,43]. However, Jung et al. found no differences in intracortical inhibition and impaired long-term potentiation-like plasticity with reduced excitatory synaptic connectivity in participants with without ID autism and Asperger syndrome [44]. Besides, evidence for short interval cortical inhibition (SICI) deficits among those ASD participants who had experienced early language delay, suggesting that GABA may be implicated in an ASD subtype [45]. Thus, regarding the critical question that whether the brain of ASD individuals is under-excited or over-excited and needs to be activated or inhibited, more robust evidence is necessary.

Commonly, the low frequency rTMS and continuous theta burst stimulation (cTBS) are considered to show inhibition on the cortex, while high frequency rTMS or intermittent theta burst stimulation (iTBS) show excitation effect on the cortex. However, recently meta-analysis illustrates that iTBS on the motor cortex was found to increase motor evoked potential (MEP) with no effect on short-interval intracortical inhibition (SICI) or intracortical facilitation (ICF), while cTBS decreased MEP and short-interval intracortical inhibition with no effect on intracortical facilitation [46]. Moreover, TBS efficacy is contributed to several factors, including the number of pulses, frequency of stimulation and brain-derived neurotrophic factor polymorphisms. Additionally, only one of the 17 studies provides the theoretical consideration for selecting the TMS mode [21] based on evidence of an animal research, in which low frequency rTMS (inhibitory protocol) has been shown to ameliorate autistic-like behaviors in rats by restoring E/I imbalance through enhancing local cortical inhibition [47]. Majority of studies apply the TMS mode according to treatment efficiency on clinical and behavior outcomes from depression [25] and other neuropsychological disease [14,23]. Thus, the intervention effects of inhibition or excitation of different TMS mode and different parameters on various brain regions are far from clear.

### 4.3 Stimulation location method

The determination of optimal stimulation parameters is essential for TMS in ASD treatment. Currently, the accuracy of the 5 cm method for locating the DLPFC has been questioned in several studies. Notably, a large trial found that the 5 cm method missed the DLPFC target in up to one-third of patients [48]. Similarly, another study reported that only 7 out of 22 subjects successfully located the DLPFC using this method [49]. Additionally, Ahdab et al. (2010) observed an average difference of 2 cm between the 5 cm rule and MRI-guided site for locating the DLPFC. Despite its limited accuracy, the 5 cm method still prevailed for its simple operation, good subject compliance, and low cost. Consequently, reducing the cost and complexity of the accurate positioning method is of paramount importance.

In recent years, functional connections obtained from resting state fMRI have been used to guide the individualized TMS treatment of neuropsychiatric conditions, and the stimulation targets are accurately located through the strongest functional connections between the deep effective brain area and the cortical brain area, thus effectively improving clinical symptoms [50,51]. Specifically, the deep effect-brain regions of the neuropsychiatric conditions were first identified, and the cortical locations with the strongest functional connections were then targeted for TMS stimulation.

### 4.4 Stimulus location

The majority of studies focus the stimulated targets on DLPFC, as this brain region plays a pivotal role in social, cognitive, and emotional functions. Research has established links between the DLPFC and the processing of verbal/auditory and non-verbal/spatial information in working memory [[52], [53], [54]]. Furthermore, magnetic resonance imaging has revealed dysfunction in the anterior cingulate cortex and DLPFC in ASD [55]. Studies have consistently demonstrated a correlation between the gamma oscillations and social deficits. Modulating gamma oscillations, particularly in the DLPFC, has been associated with improvements in cognitive performance [56,57].

The IPL was another commonly used superficial stimulation cortical target for the treatment of ASD [14,15]. The IPL belongs to the default mode network (DMN) and is involved in theory of mind (ToM) and social communications [[58], [59], [60]]. Furthermore, the IPL displayed abnormal activation in false-belief tasks according to meta-analysis [61], which reflected the deficits in ToM, impairing the individuals' ability of correctly distinguishing others’ beliefs from self-beliefs through mental/emotional states [62]. More importantly, the promising in alleviating social related symptoms has been demonstrated when IPL was used as stimulation target cortex [14]. Thus, stimulating the IPL may lead to the alleviation of ASD-related ToM deficits and social functioning symptoms.

According to a recent consensus statement for TMS for ASD, three cortical sites are generally agreed in particular: (1) the right inferior frontal gyrus targeting social impairments and communicative deficits, (2) the right temporoparietal junction/posterior superior temporal sulcus targeting theory of mind, social comprehension, and attention, and (3) the left dorsolateral prefrontal cortex targeting comorbid depressive disorder and executive dysfunction [63]. These brain regions may be selected according to the abnormalities from functional magnetic resonance imaging (fMRI) [[14], [15], [16],^23^,^32^,^39^] and modulating gamma oscillation to improve clinical symptoms [31]. Moreover, neuro-biomarker changes depicted with fMRI are increasingly popular to investigate the intervention mechanisms of TMS in depression [[64], [65], [66], [67]] rather than the E/I theory. Collectively, the theoretical considerations for selecting TMS mode and stimulation site are becoming diversified.”

### 4.5 Therapeutic effect

The evaluation of treatment effect mainly relies on ABC, ADOS, ADIR and other scales to evaluate the core characteristics of stereotyped behavior, repetitive behavior and language and social behavior. However, the inconsistent scales used by the researchers and different levels of study bias make it difficult to interpret prior significant results and confirm the effectiveness of treatment based on current literatures. There were also fewer reports of changes in the score of core symptoms, less than half of those reported for stereotyped and repetitive behaviors, and less than one-third of those reported for verbal and social behaviors. In addition to the scale, researchers also use the Wisconsin Card Sorting Test, Stroop test, Yale Brown Obsessive Compulsive Scale, Behavioral Rating Inventory for Executive Function, oddball task with rare illusory Kanizsa, and spatial working memory, to evaluate the therapeutic effect of TMS [16,17,29,39]. In addition to core symptoms, researchers also looked at ASD mood and sleep [20].

According to the priority settings outlined on the James Lind Alliance website (https://www.jla.nihr.ac.uk/priority-setting-partnerships/autism/top-10-priorities/), the hierarchy of intervention objectives for ASD, ranging from high to low priority, includes improving mental health or reducing mental health issues, refining communication/language abilities, alleviating anxiety, treatment/management of challenging behaviors, and enhancing social skills. Thus, further development of TMS interventions can also be guided by the aforementioned priorities.

### 4.6 Limitations

This systematic review is confined to articles and studies published between 2018 and 2023. Currently, investigations of stimulation regions other than DLPFC are limited. As a consequence, comparing and validating the efficacy of TMS intervention for improving autism symptoms in these target regions become challenging. Furthermore, the objective measurement methods employed in the included studies were constrained to EEG, leading to an incomplete recording of patients' changes. As a result, a comprehensive and in-depth understanding of the treatment outcomes was not achievable. Irrespective of the TMS mode, whether utilizing neuroimage navigation or EEG cap localization, the direction and angle of the stimulation coil have not received sufficient attention in current studies. However, it is crucial to consider that the angle and orientation of the coil can significantly influence the direction and efficacy of the magnetic field. Therefore, future research should address and clarify the optimal direction and angle of the stimulation coil to maximize the efficacy and safety of transcranial magnetic stimulation in treating ASD. Furthermore, TMS treatment for young children with ASD is an evolving application, but its long-term effects on the developing brain necessitate further study.

## 5 Conclusion

In general, articles utilizing TMS to treat ASD since 2018 have exhibited obvious diversity in risk of bias, stimulation sites, stimulation parameters. Recent findings generally indicate that TMS has positive effects on stereotypical behavior, repetitive behavior, verbal and social aspects of ASD, leading to overall improvement across all scale scores post-intervention. To strengthen the evidence base, future research should prioritize randomized, double-blind, sham-controlled trials. Additionally, to more comprehensively assess the efficacy of TMS treatment, it is essential to incorporate changes in physiological markers alongside routine behavioral evaluations.

## Ethics declarations

Review and/or approval by an ethics committee was not needed for this study because [This article is a systematic review and does not involve animal or human experiments].

Informed consent was not required for this study because [This study is a systematic review and does not require informed consent].

## Funding

This work is supported by Key-Area 10.13039/100006190Research and Development Program of Guangdong Province (2019B030335001), Pioneer and Leading Goose R&D Program of Zhejiang (No.2023C03002), 10.13039/501100017599Zhejiang Science and Technology Project (No.2023RC062) and Collaborative Innovation Center of Hebei Province for Mechanism, Diagnosis and Treatment of Neuropsychiatric Diseases.

## Data availability statement

Data available on request from the authors.

The data that support the findings of this study are available from the corresponding author, [Wen-Qiang Dong], upon reasonable request.

## CRediT authorship contribution statement

**Li-Xia Yuan:** Writing – review & editing, Validation, Resources, Funding acquisition, Conceptualization. **Xing-Ke Wang:** Writing – review & editing, Visualization. **Chen Yang:** Supervision, Resources. **Qiu-Rong Zhang:** Visualization, Resources. **Sheng-Zhi Ma:** Resources, Investigation. **Yu-Feng Zang:** Writing – review & editing, Validation, Funding acquisition, Conceptualization. **Wen-Qiang Dong:** Writing – review & editing, Writing – original draft, Resources, Methodology, Investigation, Conceptualization.

## Declaration of competing interest

The authors declare the following financial interests/personal relationships which may be considered as potential competing interests:Li-xia Yuan reports article publishing charges was provided by Special Project for Research and Development in Key Areas of Guangdong Province. If there are other authors, they declare that they have no known competing financial interests or personal relationships that could have appeared to influence the work reported in this paper.
